# Emission properties of Ga_2_O_3_ nano-flakes: effect of excitation density

**DOI:** 10.1038/srep42132

**Published:** 2017-02-08

**Authors:** G. Pozina, M. Forsberg, M. A. Kaliteevski, C. Hemmingsson

**Affiliations:** 1 Department of Physics, Chemistry and Biology (IFM), Linköping University, S-581 83 Linköping, Sweden; 2 St-Petersburg Academic University Khlopina 8/3, 194021, St-Petersburg, Russia; 3 ITMO University, Kronverkskiy pr. 49, 197101 St. Petersburg, Russia

## Abstract

In the quest of developing high performance electronic and optical devices and more cost effective fabrication processes of monoclinic β-Ga_2_O_3_, new growth techniques and fundamental electronic and optical properties of defects have to be explored. By heating of dissolved metallic Ga in HCl in a NH_3_ and N_2_ atmosphere, nano-flake films of monoclinic *β*-phase Ga_2_O_3_ were grown as confirmed by XRD. From optical measurements, we observe two strong emissions. A red band peaking at ~2.0 eV and a UV band at ~3.8 eV. The band at ~2.0 eV is attributed to donor-acceptor pair recombination where the donor and acceptor level is suggested to be related to V_O_ and nitrogen, respectively. By studying the dependence of the intensity of the UV band at 3.8 eV versus excitation density, a model is suggested. In the model, it is assumed that local potential fluctuations forming minima (maxima), where the carriers would be localized with a summarized band offset for conduction and valence band of 1 eV. The origin of the fluctuations is tentatively suggested to be related to micro-inclusions of different phases in the film.

During the last 20 years there has been a rapid development of wide band gap semiconductors such as SiC and GaN, which permitted novel types of optoelectronic applications for efficient solid state lightning, blue-UV semiconductor lasers, and power diodes and transistors that demonstrate significantly higher performance while demanding less energy than silicon-based devices^1^. However, one of the major bottlenecks with SiC and III-nitrides is related to very complicated and expensive processes for fabrication of large area bulk substrates^2^,^3^,^4^. This explains a huge recent interest to another wide band gap semiconductor, gallium oxide (Ga_2_O_3_), since it is possible to produce rather cheap high-quality single crystal substrates from a melt, which is a significant advantage for industrial applications. Additionally, monoclinic *β*-Ga_2_O_3_ turns out to be an ideal material for future power devices in ultra-high voltage switching applications. The break down field is expected to be around 8 MV/cm, which is larger than for both GaN and SiC^5^.

*β*-Ga_2_O_3_ is the most stable phase having a large band gap of ~4.8 eV^6^, making it one of the most important transparent conductive oxides (TCO) for fabrication of transparent electrodes in smart windows technology, photovoltaic devices, liquid crystal displays, chemical sensors and light emitting diodes. For the use as transparent electrodes, Ga_2_O_3_ films could have polycrystalline structure, which makes the synthesis processes less demanding and significantly cheaper than, for example, epitaxial growth, but the material shows usually a high level of point defects. Additionally, Ga_2_O_3_ thin films can be used as an n-type semiconductor layer in high-efficiency heterojunction solar cells^7^. There are different methods to synthesize Ga_2_O_3_ films such as, for example, physical evaporation^8^, arc-discharge^9^, carbothermal reduction^10^, chemical vapor deposition (CVD)^11^ and microwave plasma^12^. Although, the structural properties studied rather intensively, the fundamental knowledge of luminescence and point defect properties in Ga_2_O_3_ is very poor, which requires additional extensive studies of this material.

Thus, in this work we present results of optical study of the *β*-Ga_2_O_3_ nanoflake films showing bright UV and red emission bands at ~3.8 and ~2.0 eV, respectively. The 3.8 eV transition is less known and the origin of it is still unidentified. Possible recombination mechanisms based on luminescence properties obtained by cathodoluminescence (CL) and time-resolved photoluminescence (PL) are suggested for both emission lines.

## Results

**Figure 1:**
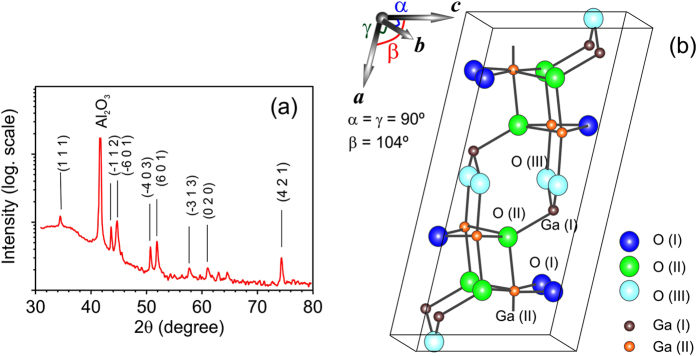
(**a**) Typical XRD spectrum shows peaks which are identified for *β*-Ga_2_O_3_. (**b**) Schematic draw of the unit cell of *β*-Ga_2_O_3_. There are two nonequivalent Ga sites marked by small spheres Ga (I) (brown) and Ga (II) (orange). There are three nonequivalent O-sites marked by big spheres O (I) (blue), O (II) (green) and O (III) (turquoise).

The obtained Ga_2_O_3_ films were crystalized in β-phase, which was confirmed by XRD measurements. A typical XRD pattern is presented in Fig. 1(a), where different diffraction peaks can be well attributed to the monoclinic β-phase Ga_2_O_3_ (PDF # 41-1103). The luminescence properties of the *β*-Ga_2_O_3_ with its wide-band gap and low crystal symmetry are rather complex and are the subject of intensive studies. Monoclinic base-centered structure of *β*-Ga_2_O_3_ shown in Fig. 1(b) has C2/m space group with the following lattice parameters: *a* = 12.23 Å, *b* = 3.04 Å, *c* = 5.80 Å and with the angle β between axes *a* and *c* of ~104° (other angles α = γ = 90°). In the unit, there are two inequivalent position for gallium atoms [indicated as Ga(I) and Ga(II)] and three - for oxygen atoms [marked by O(I), O(II) and O(III)]. As a result, impurities and structural point defects such as vacancies and interstitials build a large variety of different energy levels within the band gap that, in turn, leads to a complex optical emission spectrum with several peaks from UV to red region.

**Figure 2:**
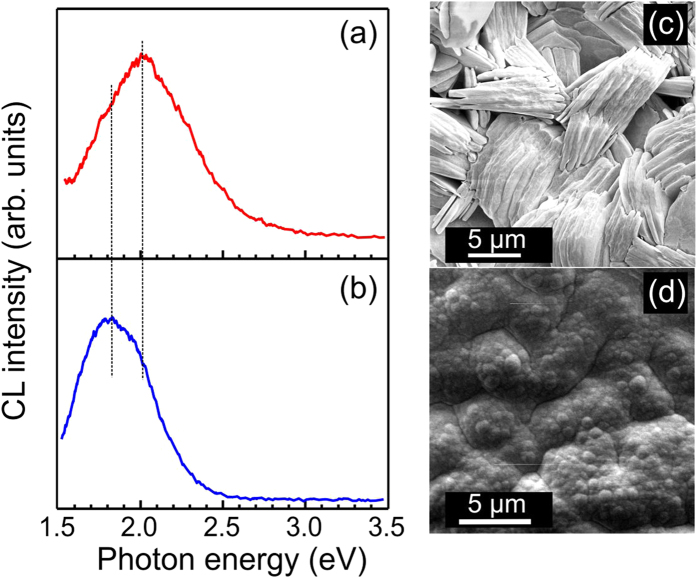
Typical CL emission measured for the Ga_2_O_3_ film in cross-section (**a**) and top-view (**b**) geometry with corresponding CL images shown in (**c**) and (**d**), respectively.

CL measurements reveal that the Ga_2_O_3_ film luminescence is dominated under standard excitation conditions by a broad emission band in the visible region as presented in Fig. 2. Normalized CL spectra in Fig. 2(a) and (b) are detected from the areas displayed in SEM images in Fig. 2(c) and (d), respectively. We have observed that the CL spectrum taken at the cross-section of the film shows a broader band with the peak position at ~2.0 eV, i.e. blue shifted about 0.2 eV compared to the emission peaked at ~1.8 eV (though the shoulder at ~2.0 eV is also presented), which was acquired in the top-view geometry from the same sample. We note here that almost no UV emission could be detected during these measurements.

**Figure 3:**
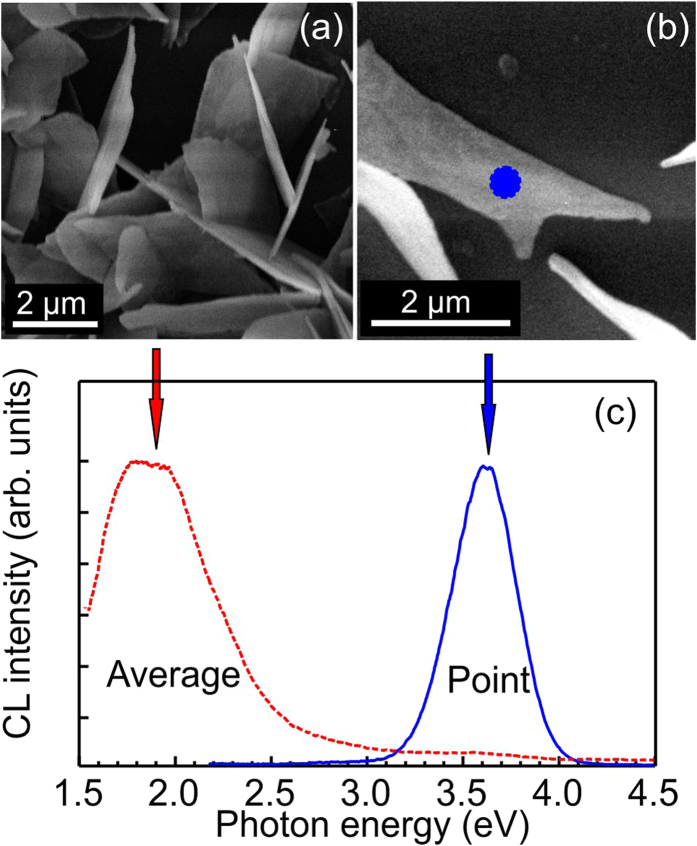
(**a**) SEM image of Ga_2_O_3_ nanoflakes. (**b**) SEM image of a single nanoflake. (**c**) CL spectrum shown by the red dashed line is acquired from the sample area presented in (**a**) and CL spectrum shown by the blue solid line is acquired from the single nanoflake when electron beam was focused to the point as marked in (**b**). Spectra are normalized.

However, when the electron beam is focused to a point and, consequently, the excitation density increases, the CL spectrum is dramatically changed; namely, a strong UV emission line appears, while the red emission has almost completely vanished. This phenomena is illustrated in Fig. 3, where we compare the CL spectrum (red dashed line) taken in scanning mode from several nanoflakes shown in Fig. 3(a) with the spectrum for one nanoflake (blue solid line) measured from the point indicated at the SEM image in Fig. 3(b). In the visible band, two features at 1.8 and 2 eV can be resolved. The UV band has one maximum at ~3.7 eV though the line shape is slightly asymmetrical.

**Figure 4:**
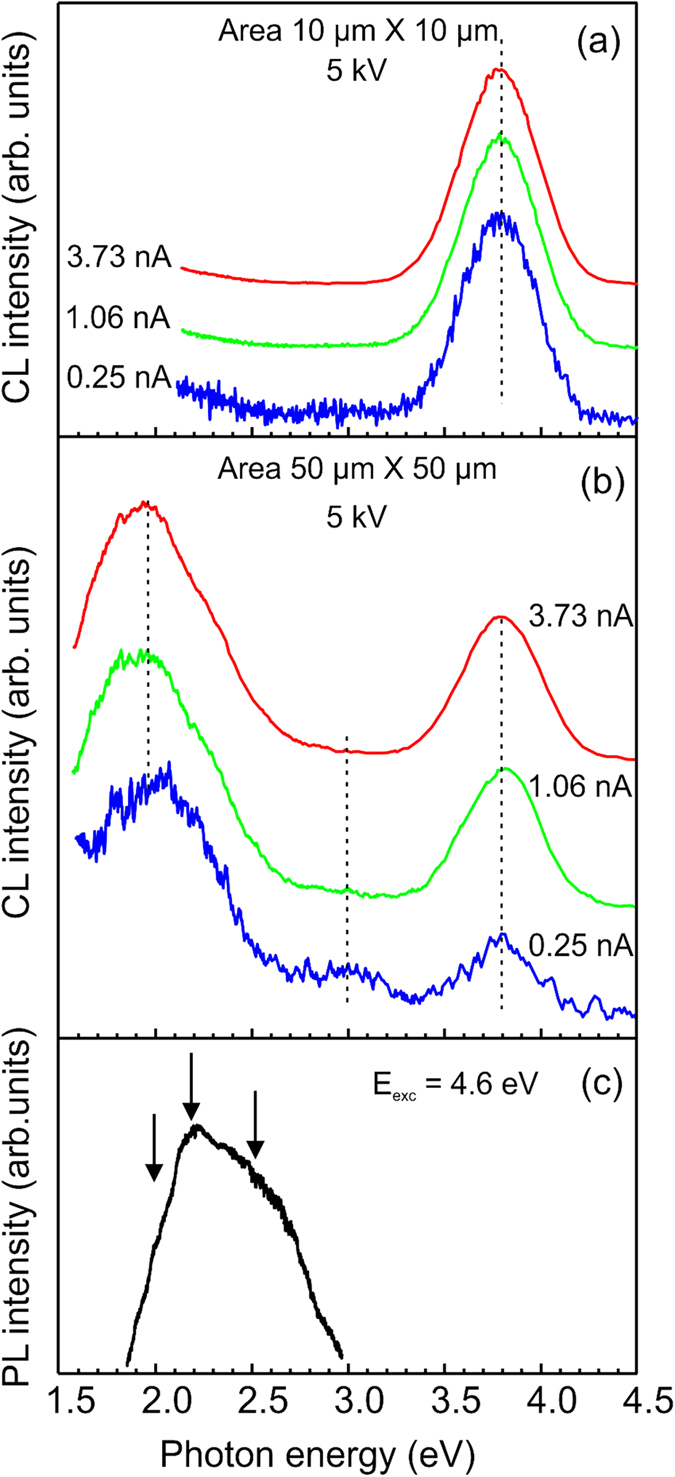
(**a**,**b**) Normalized CL spectra measured at different current of the electron beam in a scanning mode. The acquisition area was (**a**) 10 μm × 10 μm and (**b**) 50 μm × 50 μm, respectively. The beam current is indicated for each spectrum. Spectra are shifted vertically for clarity. (**c**) PL spectrum from the same sample under excitation with the laser wavelength of 266 nm (4.6 eV).

The increase of the electron beam current can have similar effect even if the measurements are done in a scanning mode. A definitive conclusion could not be done because the maximum current was limited by instrument to 3.73 nA. On the other hand, it seems that reducing the scan area is a more efficient way of excitation of the UV emission. The CL data are presented in Fig. 4(a) and (b) for three different beam currents for the case when CL spectra are collected from the smaller and larger scan areas of ~10 μm × 10 μm and ~50 μm × 50 μm, respectively. To minimize the contribution of possible inhomogeneity of the film, the sample position between measurements in (a) and (b) was not changed, only the magnification was different. We have examined several points and the effect was similar, i.e. at the higher excitation densities we observe an enhancement of the UV band compared to the red emission. Thus, it is clear that the excitation conditions influence the shape of the CL emission. Data in Fig. 4(a) are similar to the case of point excitation when the UV line dominates CL spectrum even at the lowest current of 0.25 nA. For the larger raster, see in Fig. 4(b), three emission bands, UV, blue and red, can be detected with maxima at ~3.8, ~3.0 and ~2.1 eV, respectively. However, now the red emission dominates the CL spectrum at all beam currents although the relative intensity of the UV band slightly increases with the beam current. The weakest band at ~3.0 eV (so-called blue emission) is not resolved under excitation by higher electron beam currents. Another observation is related to the spectral shift caused by excitation conditions. The maximum energy of the UV band (i.e. 3.8 eV in scanning mode) is shifted compared to the peak position of 3.7 eV observed under measurements in focused mode (Fig. 3(b)). It is interesting to compare CL spectra with PL spectrum shown in Fig. 4(c), since excitation densities are much lower in PL than in CL, where one electron with the energy of 5 keV can create several hundred electron-hole pairs. Since the PL excitation was done using a laser wavelength of 266 nm (or 4.66 eV), i.e. inside the *β*-Ga_2_O_3_ band gap, only intraband transitions are possible. We have observed a weak broad emission with a maximum at ~2.2 eV and with additional features at ~1.9 and ~2.5 eV, respectively. As mentioned, taking into account the lower excitation densities in PL compared to CL, the PL peak position is in line with the observed trend in CL under excitation using lower electron beam currents.

**Figure 5:**
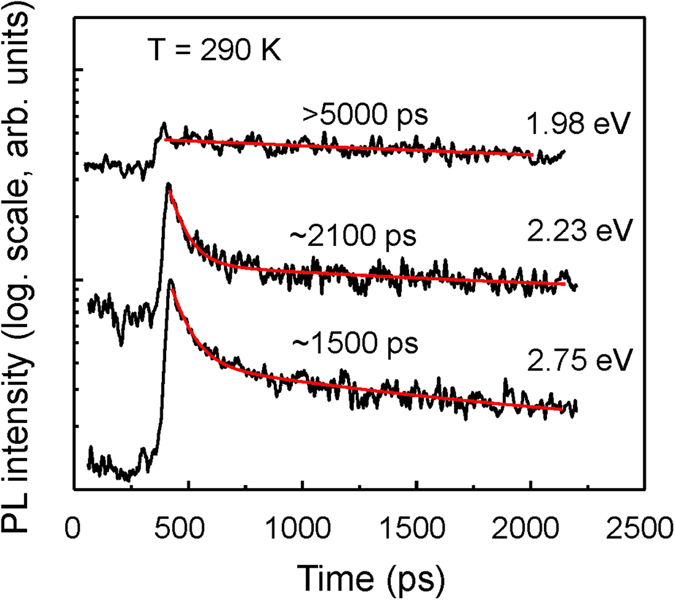
PL decay curves (black lines) measured at different photon energies of 1.98, 2.23 and 2.75 eV. Fitting using bi-exponential decay law is shown by red lines. The long time constant is indicated for each curve.

Temporal behavior of the red band was analyzed by time-resolved PL. We have found that PL recombination time strongly depends on the wavelength. Thus, for the high energy side the PL decay curves obey a bi-exponential law with fast and slow components, while for the low energy shoulder, the PL intensity decays rather slowly following a single exponential decay function. The examples of the PL decay curves are presented in Fig. 5 by black lines. Fitting using a bi-exponential decay law is also shown (red lines). For the peak position and for the higher energy side of the emission band, the fast recombination time is ~80 ps. The slow component becomes more dominant for the lower photon energies and for the feature at 1.98 eV the PL decay is characterized by only a slow recombination time exceeding 5 ns.

## Discussion

Though the luminescence in *β*-Ga_2_O_3_ has been previously discussed in literature, there are still difficulties with a doubtless assignment of different emission bands. Depending on samples growth conditions and doping, there have been reported several broad PL peaks with positions ranged in the energy interval of ~1.7–3.8 eV^13^,^14^,^15^,^16^,^17^,^18^,^19^,^20^,^21^. Below we summarize briefly some assignments of the luminescence in *β*-Ga_2_O_3_. Thus, Zhou *et al*.^13^ observed in the *β*-Ga_2_O_3_ nanostructures having a crystalline core and an amorphous shell the UV and red emission bands at ~3.4 and ~1.78 eV, respectively, both characterized by a shorter recombination time; on the other hand, the blue emission at ~2.7–3.0 eV and the green band at 2.35 eV demonstrated longer lifetimes. The origin of the blue band have been assigned in agreement with other reports^14^,^15^,^16^,^17^ to the recombination of carriers trapped by the oxygen and gallium vacancies forming deep donor and acceptor states, respectively. In contrary, the UV and red lines have been related to the presence of a subband formed by amorphous Ga_2_O_3_, which is in contradiction with results reported by other groups^14^,^15^,^16^. The UV luminescence (~3.45 eV), with a reference to the early paper of Harwig *et al*.^14^, is commonly assigned to a recombination of self-trapped excitons considering that this emission is absent under excitation below the band gap. The green emission (~2.3 eV) was usually observed in samples doped, for example, by Si^17^; however, Villora *et al*. concluded that this band is associated with self-trapped or bound excitons^18^. The red band also detected in single crystal *β*-Ga_2_O_3_^19^ has been related to electron–hole recombination via the vicinity donors and acceptors^19^. However, it was also reported that the appearance of the red band at ~1.78 eV can be correlated with nitrogen doping^20^.

Intrinsic *β*-Ga_2_O_3_ is unintentionally n-type doped, though the residual donor concentration is very low (~10^13^ cm^−3^) and the origin of the n-type conductivity is still under debate. Binet *et al*.^16^ suggested that oxygen vacancies (V_O_) play a role of donors with ionization energy about 40 meV. This has been questioned by Onuma *et al*.^6^ after electrical measurement studies correlated with CL. They suggested that V_O_ acts as a deep level with an activation energy of ~1.0 eV. From theoretical calculations, it has been ruled out that V_O_ is a shallow donor in intrinsic *β*-Ga_2_O_3_. It was suggested that Si can be used as a shallow donor^22^. Recently, it was shown using density function theory (DFT) calculations that C–H groups are deep acceptors in *β*-Ga_2_O_3_ and compensate for the intentionally introduced donors^23^. Clearly, intrinsic point defects and impurities affect emission spectra in *β*-Ga_2_O_3_, however, it seems that a correct identification is complicated.

While our samples have not been specifically doped, common impurities such as Si, N, H and C could be presented. We have observed only two strong emissions: the red band peaking at ~2.0 eV and the UV band at ~3.8 eV. The last one is almost unknown except it was mentioned in ref. 13. Based on our investigations, the red emission band peaking at ~2.0 eV is likely due to donor-acceptor pair (DAP) recombination. The energy of the DAP emission depends on the distance between donor and acceptor meaning also that the recombination time for such emission is depending on photon energy. For the carrier pairs separated by large distances (and recombining emitting lower energy photons), the recombination time will be longer. We have observed those characteristics for the red band at ~2.0 eV. The energy of the DAP emission is written as:


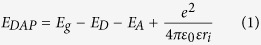


Here *r*_*i*_ is the donor-acceptor distance, the band-gap energy *E*_*g*_ = 4.8 eV, *E*_*D*_ and *E*_*A*_ are donor and acceptor levels, respectively. If the energy of donor (i.e. V_O_) is *E*_*D*_ = ~1.0 eV^6^, then the acceptor levels energy can be estimated from Eq. (1) to *E*_*A*_ = 1.8 eV, taking into account the CL peak position of ~2.0 eV for the red emission. The acceptor levels can likely be associated with nitrogen, since our samples were annealed in ammonia and nitrogen gases. Indeed, the energy dispersive X-ray spectroscopy (EDS) composition analysis of the Ga_2_O_3_ nano-flake film has shown a presence of nitrogen with concentrations between 0.5 and 1.8 Wt %. Places on the film with lower nitrogen concentrations demonstrated the red emission peaking at 2.0–2.1 eV (Fig. 2(a)), while areas with high nitrogen concentration have shown red band centered at ~1.8–1.9 eV (Fig. 2(b)).

**Figure 6:**
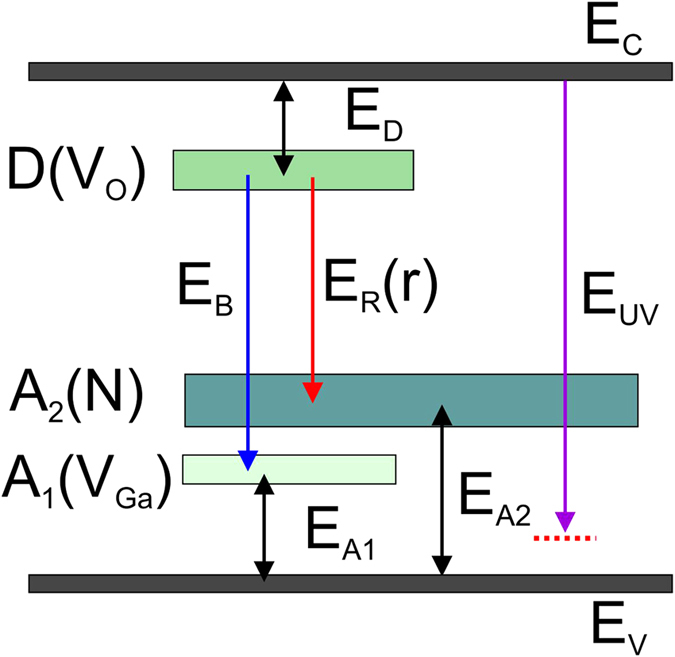
Schematic drawing of the band diagram in Ga_2_O_3_. Ec and Ev are the conduction and the valence band, respectively. D(V_O_) indicates a donor band formed by oxygen vacancies, A_2_(N) and A_1_(V_Ga_) denote acceptor bands built by nitrogen impurities and gallium vacancies, respectively.

DFT calculations show that nitrogen forms deep acceptor levels at about the center of the band gap^24^, which is in qualitative agreement with our finds. Song *et al*.^25^ indicated that there is a shift of the red emission to the lower energies with increasing nitrogen concentration. This is in line with our observations as shown in Fig. 2 for the CL measurements taken at the film surface, which was more exposed to nitrogen and to ammonia during annealing. Thus, we suggest that the red emission *E*_*R*_(*r*) at ~2.0 eV corresponds to the DAP recombination, where donors are formed by oxygen vacancies, while acceptor levels are formed by nitrogen impurities. See Fig. 6 showing schematic representation of the band diagram in Ga_2_O_3_, where levels D(V_O_) and A_2_(N) have energies *E*_*D*_ = ~1.0 eV and *E*_*A2*_ = 1.8 eV from corresponding band edges, respectively.

The assignment of the UV emission at ~3.8 eV is more complicated. With an increasing excitation densities, a strong enhancement of the UV emission compared to the red line is detected in our samples. Additionally, the shift of the peak position to lower energies (~0.1 eV) is observed for the highest excitation density, when the electron beam is focused to one point (Fig. 3(b)). It is understandable that as more free electrons are created, the DAP recombination (i.e. red emission) decreases since the number of donor and/or acceptor levels is limited, while free carrier recombination should increase. It is known that the Mott transition can occur at high carrier densities leading to the band gap renormalization with corresponding red shift of the band-to-band transition^26^,^27^, which can explain the 0.1 eV shift to the lower energies for the UV line. However, the observed peak position of ~3.8 eV (corresponds to E_UV_ in Fig. 6) is below the band gap energy in *β*-Ga_2_O_3_ by ~1 eV. Such difference can hardly be explained by only the band gap narrowing, which is usually in the range of several meV, occurring at high excitation density or high doping^26^,^27^,^28^,^29^.

**Figure 7:**
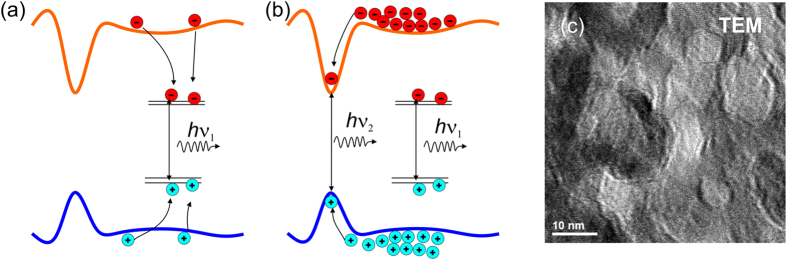
Schematic draw of the band profile fluctuations. Electrons (red circles) and holes (blue circles) undergo different recombination process at (**a**) low and (**b**) high excitation densities. (**c**) HRTEM image of the Ga_2_O_3_ nano-flake film.

We assume now that the potential is not uniform and that there are local potential minima in the material. The CL data can be understood in the frame of processes schematically illustrated in Fig. 7. Under low excitation, created electrons (holes) are firstly relaxed to the band edges and trapped by the defect levels inside the band gap and then carriers recombine producing a photon with the energy *hν*_*1*_ corresponding to the red emission as shown in Fig. 7(a). The recombination via the defect levels inside the band gap is the main recombination channel, which explains our observations that the red emission band is dominating in this case. When the excitation density is high, the defect traps are filled as depictured in Fig. 7(b). Consequently, the relative contribution of radiative transitions via the traps will be reduced compared to the band-to-band recombination. However, the peak energy of 3.8 eV for the UV line is lower than the band gap energy meaning that the transition origin is not directly related to the band-to-band recombination in *β*-Ga_2_O_3_. Likely, the potential profile is not uniform within the film and there are local potential fluctuations forming minima (maxima), where the carriers would be localized with a summarized band offset for conduction and valence band of 1 eV. It is not necessary that potential minima for electrons would correspond to the potential maxima for holes indicating that electron and hole recombination (with photon energy *hν*_*2*_) in such indirect potential wells can be much less efficient compared to the emission via the defect levels. However, the UV luminescence starts to dominate CL spectra at higher excitation densities while the defect luminescence starts to saturate. This model describes rather reasonable the observed experimental data, though still the origin of the potential fluctuations should be determined. Tentatively, we suggest that such potential fluctuations can be caused by micro-inclusions of different phases as it was pointed by Zhou *et al*.^13^ and/or structural defects in similarity with stacking faults building a quantum well-like formation with characteristic emission energy as observed, for example, in GaN^30^,^31^. High resolution transmission electron microscopy (HRTEM) images (see example in Fig. 7(c)) have shown a rather complex structure of nano-flakes. A number of different grains and domains of several nm in size can be distinguish in TEM images. It is clear that such complicated structure can give a rise to potential fluctuations.

In summary, *β*-Ga_2_O_3_ nano-flake films grown by heating of dissolved metallic Ga in HCl in a NH_3_ and N_2_ atmosphere at 600 °C for 30 min have been studied by XRD, SEM, CL and time-resolved PL. XRD measurements obtained for the nano-flakes have shown presence of monoclinic *β*-phase Ga_2_O_3_.

Optical measurements revealed two strong broad emission peaks. A red band peaking at ~2.0 eV and a UV band at ~3.8 eV. Due to the behavior of the recombination time with increasing photon energy and the peak position of the band at ~2.0 eV, we suggest it is related to donor-acceptor pair (DAP) recombination where V_O_ is the donor. The acceptor level could be related with nitrogen, since the nano-flakes were grown in a nitrogen containing ambient.

A model of the origin of the UV band at ~3.8 eV was suggested. In the model, it is assumed that local potential fluctuations form minima (maxima), where the carriers would be localized with a summarized band offset for conduction and valence band of 1 eV. However, the origin of the potential fluctuations is still not verified; tentatively, we suggest that they can be caused by micro-inclusions of different phases as it was pointed by Zhou *et al*.^13^.

## Methods

For growth of polycrystalline Ga_2_O_3_ nanoflake films we used the following procedure: at first, metallic Ga was dissolved in HCl mixed with deionized water. Then, the gallium chloride solution was drop deposited on the substrates and placed in the oven for annealing at 600 °C in ammonia (NH_3_) and nitrogen gas flow for 30 min in the halide vapor phase epitaxy (HVPE) reactor. As substrates, we used (0001) sapphire of optical quality, which is convenient due to its chemical stability and due to the absence of the luminescence signal simplifying optical characterization of Ga_2_O_3_.

X-ray diffraction (XRD) scans were performed in a Philips 1820 Bragg-Brentano diffractometer using Cu-Kα radiation. CL measurements were done using a standard Leo 1500 Gemini scanning electron microscope (SEM) combined with the MonoCL4 system. CL images were acquired using a Peltier-cooled GaAs photomultiplier tube. Spectral acquisition was done with a CCD detector using a 150 l/mm diffraction grating. The spectral resolution under the experimental conditions was ~10 nm. All measurements were done at room temperature and at acceleration voltage of 5 kV. Standard electron beam current was ~0.25 nA, in other cases it is indicated separately. Chemical composition was analyzed using energy dispersive X-ray spectroscopy (EDS) equipped with an Oxford LINK ISIS system with a Ge detector. TEM imaging was done with a high resolution FEI Tecnai G2 200 keV FEG microscope. Nano-flakes were removed from the substrate, suspended in ethanol and then placed on the 200 mesh copper grid. For detection of time-resolved photoluminescence we have used a Hamamatsu synchroscan streak camera with a temporal resolution of 20 ps. The available excitation wavelength of 266 nm (i.e. below the band gap of *β*-Ga_2_O_3_) was a third harmonics of the Ti-sapphire femtosecond pulsed laser with a frequency of 75 MHz.

## Additional Information

**How to cite this article**: Pozina, G. *et al*. Emission properties of Ga_2_O_3_ nano-flakes: effect of excitation density. *Sci. Rep.* **7**, 42132; doi: 10.1038/srep42132 (2017).

**Publisher's note:** Springer Nature remains neutral with regard to jurisdictional claims in published maps and institutional affiliations.
